# Novel Use of Intraoperative Fluoroscopy in an Era of ICG for Complex Laparoscopic Cholecystectomy

**DOI:** 10.1055/s-0040-1721432

**Published:** 2021-02-28

**Authors:** Jignesh A. Gandhi, Pravin H. Shinde, Sadashiv N. Chaudhari, Amay M. Banker

**Affiliations:** 1 Department of GI and Laparoscopic Surgery, Global Hospital, Mumbai, Maharashtra, India; 2 Department of General Surgery, Seth G.S. Medical College and KEM Hospital, Mumbai, Maharashtra, India

**Keywords:** fluoroscopy, gall bladder, CBD stenting, laparoscopic cholecystectomy, gangrenous cholecystitis

## Abstract

**Background** Laparoscopic cholecystectomy (LC) is increasingly being used as a first-line treatment for acute cholecystitis. Bile duct injury (BDI) remains the most feared complication of the minimally invasive approach specially in cases with an inflamed calots triangle. While use of indocyanine dye (ICG) to delineate biliary anatomy serves to reduce BDI, the high cost of the technology prohibits its use in the developing world. We propose a novel use of common bile duct (CBD) stenting preoperatively in cases of cholecystitis secondary to choledocholithiasis as a means of identification and safeguarding the CBD.

**Methods** A retrospective review was conducted on 22 patients of Grade 2 or Grade 3 cholecystitis who underwent an early LC at our institution. All patients were stented preoperatively and the stent was used for a much-needed tactile feedback during dissection. A c-arm with intraoperative fluoroscopy was used to identify the CBD prior to clipping of the cystic duct.

**Results** The gall bladder was gangrenous in all the cases while two cases had evidence of end organ damage. This innovative use of CBD stenting allowed us to correctly delineate biliary anatomy in all of the cases and we report no instances of BDI despite a severely inflamed local environment.

**Conclusion** This technique can become a standard of care in all teaching institutions in developing countries further enhancing the safety of cholecystectomy in gangrenous cholecystitis with a distorted biliary anatomy.

With over a million procedures per year, laparoscopic cholecystectomy (LC) is one of the most common surgeries performed worldwide. 1 Traditionally acute cholecystitis was a relative contraindication of the laparoscopic approach. However, as surgical techniques have improved and significant advancements have been made in minimal access technology, LC has now become the standard of care for moderate (Grade 2) cholecystitis and can be used as a straightforward procedure in severe (Grade 3) cholecystitis as well. 2 Bile duct injury (BDI) remains the most feared complication of the laparoscopic approach and the rate of BDI's increases in cases of acute cholecystitis. 3 4 5 This can have disastrous consequences for the patient and it has a significant negative impact on the quality of life even 10 years after the event. 6 7 8 9

Use of Indocyanine Green (ICG) dye and a near infrared camera to delineate the biliary anatomy has emerged as one of the ways to minimize intraoperative complications. 3 10 11 However, the high cost of the laparoscopic set up puts it out of reach for many surgeons in the developing world. Routine use of intraoperative cholangiogram in LC is controversial due to a significantly increased operating time, requirement for cannulation of the bile duct with a concomitant increase in the risk of BDI. 10

Here we describe a novel use of preoperative common bile duct (CBD) stenting and intraoperative fluoroscopy for easier identification and safeguarding of the CBD in cases of acute cholecystitis.

## Materials and Methods

A retrospective review of patients who underwent an early LC for acute cholecystitis at a referral care center catering to a population of 10 million was performed after approval from the Institutional Ethics Committee.

All patients in the 2017 to 2019 period who were diagnosed with Grade 2 or Grade 3 acute cholecystitis 12 secondary to CBD stone or sludge were included in the study. Patients who were pregnant, unfit for general anesthesia, or who had a bleeding diastasis were excluded from the study.

The data collected consisted of patient demographics such as age, sex, body mass index (BMI), and comorbidities. Perioperative data such as blood investigations, the American Society of Anaesthesiologists (ASA) score, operative time, and intraoperative blood loss were also collected.

Primary outcome variables were complications including BDI, requirement of bail out procedures, and duration of hospital stay. Patients were followed up after 1 and 6 months.

## Technical Details

### Preoperative Care

**Fig. 1:**
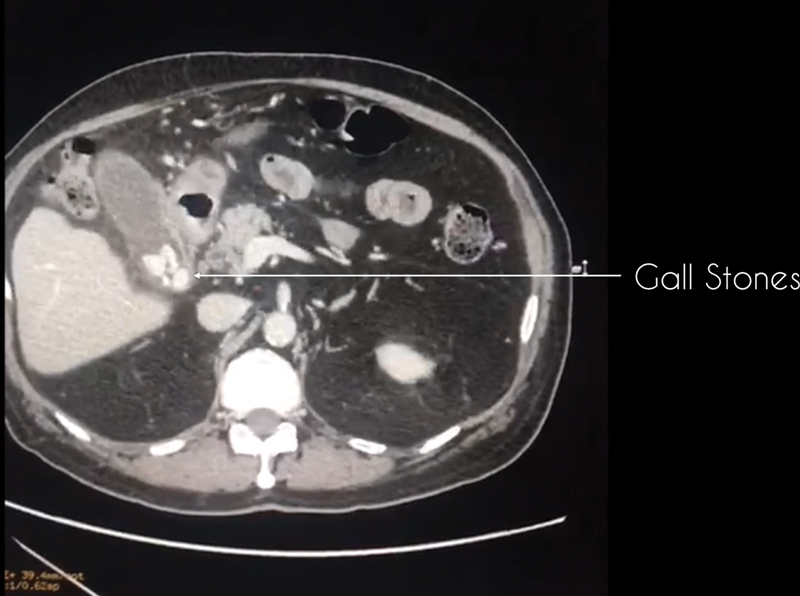
CT image showing gall stones and surround peri gall bladder fat stranding. CT, computed tomography.

**Fig. 2:**
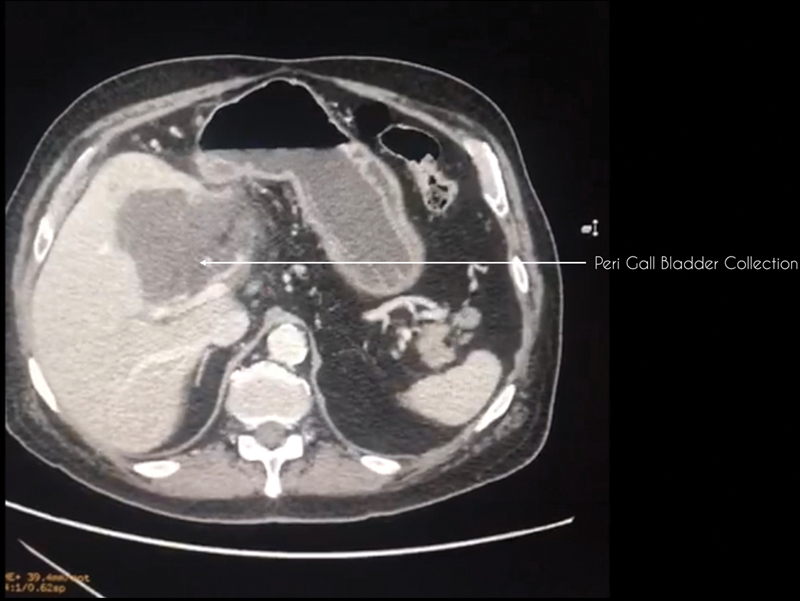
CT image showing collection in the gall bladder fossa suggestive of a perforated gall bladder. CT, computed tomography.

All patients diagnosed with moderate or severe cholecystitis are admitted to the intensive care unit for monitoring of hemodynamics. Adequate intravenous fluid infusion and electrolyte correction is ensured, and broad spectrum empirical antibiotic therapy is started as per the current recommendations. 13 An ultrasonography (US) of the abdomen is performed to look for signs of local inflammation. A computed tomography (CT) is strongly advocated to delineate the local anatomy, to look for peri gall bladder (GB) collection, evidence of gangrenous or emphysematous GB, to assess the CBD, and to look for variations in the vascular anatomy of the biliary system ( Figs. 1 and 2 ). Using an endoscopic retrograde cholangiopancreatography (ERCP) approach stenting of the CBD is done preoperatively. ERCP is performed using CO _2_ insufflation so an early LC can be planned. All patients are taken up for LC within 24 hours of the stenting.

### Surgical Technique

The patients are operated under general anesthesia using the standard four-port technique. A few technical modifications are advocated in cases of severe inflammation. A distended GB is decompressed with an 18-Gauge spinal needle and GB bile is sent for culture and antibiotic sensitivity. Dissection of the posterior leaf of peritoneum covering the neck of the GB is commenced above the Rouviere's sulcus and effective retraction of the GB is ensured to dissect the Calot's triangle and achieve the critical view of safety (CVS).

For persistent hemorrhage, hemostasis is achieved primarily by compression and excessive use of electrosurgery is avoided. An efficient suction-irrigation system is required to clear the operative field. An additional port for retraction or suction is used whenever necessary.

The operating surgeon, the staff nurse, anesthetist, and one technician are made to don personal protective equipment (PPE) consisting of a 0.5-mm lead apron with a thyroid shield. All nonessential personnel are made to stay away from the exposed area. A mobile c-arm unit is then positioned over the target area and a pulsed fluoroscopy technique is used to reduce radiation exposure. 14 This imaging helps the surgeon visualize the preoperatively placed stent and gain a better understanding of the three-dimensional anatomy of the CBD. A laparoscopic instrument is placed near the dissected Calot's triangle as a marker to understand the relationship between the CBD and the cystic duct.

**Fig. 3:**
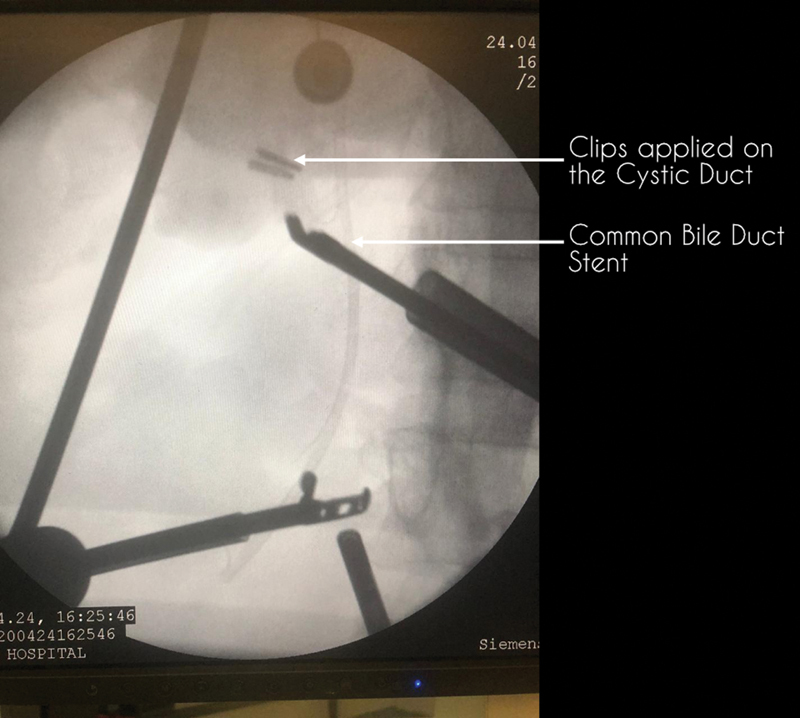
Fluoroscopic image depicting the common bile duct (CBD) stent and the cystic duct clips. The CBD can be identified easily with the help of fluoroscopic imaging and can therefore be safeguarded. CBD, common bile duct.

Once the anatomy is delineated and a radiological-assisted CVS is achieved, the cystic duct is doubly clipped and cut. If the cystic duct is dilated, a 60-mm blue endostapler or an endoloop can be used to secure the cystic duct. A repeat fluoroscopic shoot is taken to confirm the location of the clips in relation to the CBD ( Fig. 3 ). The operating staff is then made to doff the PPE and rescrub to complete the remainder of the surgery. The cystic artery is also cut between two clips and the GB is then flagged off the liver surface. A specimen retrieval bag is used to extract the GB via the epigastric port. An abdominal drain is placed into the GB fossa and the ports are removed under direct vision.

### Postoperative Care

Antibiotics are continued in the postoperative period and are modified as per culture sensitivity reports. Acetaminophen is used to offer analgesia. Patients are allowed oral sips in the evening and diet is advanced after complete resolution of postoperative ileus. Drain is removed once daily output is <20 mL. A repeat ERCP with removal of stent is done 4 weeks after the surgery.

## Results

### Demographics

A total of 22 cases were included in the study. The results are summarized in Table 1 . Majority of the cases were females. The median age in this cohort was 42 years and the mean BMI was 29.2 kg/m ^2^ . Many patients had comorbidities including diabetes mellitus, anemia, chronic obstructive pulmonary disease, hypertension, and ischemic heart disease.

**Table 1:** Patient demographics

Total patients	22
Sex	
Male	7 (32%)
Female	15 (68%)
Age, years	42 (35–49)
BMI, kg/m ^2^	29.2 (±2.86)
Comorbidities a	
Diabetes mellitus	9 (41%)
Anemia	4 (18%)
Hypertension	3 (14%)
COPD	2 (9%)
Ischemic heart disease	1 (5%)

Abbreviations: BMI, body mass index; COPD, chronic obstructive pulmonary disease.

Note: Values are listed as either number (percentage), mean (±standard deviation) or median (interquartile range).

a Comorbidities coexisted in a few patients.

Since cases with moderate or severe cholecystitis were included in the present study leukocytosis was present in all of the cases. The total bilirubin was mildly elevated with a mean value of 1.89 mg/dL probably due to compression of the CBD secondary to local inflammation. Elevation of liver enzymes was also noted ( Table 2 ).

**Table 2:** Laboratory values

Total patients	22
TLC counts/mm ^3^	19,333 (±3,599)
Hemoglobin, mg/dL	11.2 (±2.44)
ALT, U/L	97.78 (±46.66)
AST, U/L	111.33 (±36.78)
ALP, U/L	376 (±76.98)
Total bilirubin, md/dL	1.89 (±0.44)

Note: Values are listed as mean (standard deviation).

Abbreviations: ALP, alkaline phosphatase; ALT, alanine transaminase; AST, aspartate transmaninase; TLC, total leucocyte count.

### Perioperative Details

All patients were diagnosed and classified as per the Tokyo Guidelines 2018. In the present cohort, 20 cases were of moderate cholecystitis while two cases had evidence of end organ damage. There was severe local inflammation in all the cases with the GB being either gangrenous or emphysematous. 63% of the cases were ASA class II with systemic illness. The average duration of surgery was 102 minutes with an estimated blood loss of 150 mL ( Table 3 ).

**Table 3:** Operative data

Total patients	22
Grade of cholecystitis	
Grade 1	0
Grade 2	20 (91%)
Grade 3	2 (9%)
ASA score	
I	0
II	14 (64%)
III	7 (32%)
IV	1 (4%)
Blood loss, mL	150 (100–200)
Operative time, min	102 (77–127)

Abbreviation: ASA, American Society of Anaesthesiologist score.

Note: Values are listed as either number (percentage), mean (range).

Out of the 22 LC for acute cholecystitis, one case required conversion to open because of a frozen Calot's triangle and a laparoscopic fundus first approach was used as a bail out procedure in three cases. There were no instances of an ERCP-induced complications such as pancreatitis, bleeding, perforation, or cholangitis in the present study. We were successfully able to identify the CBD in all of our cases which prevented BDI in the present cohort ( Table 4 ).

**Table 4:** Postoperative data

Total patients	22
Bile duct injury	0
Bail out procedures	
Conversion to open	1 (4%)
Partial cholecystectomy	0
Fundus first approach	3 (14%)
Systemic complications	
MI	1 (4%)
UTI	2 (9%)
Visual analog scale	
< 7	15 (68%)
> 7	7 (32%)
Length of hospital stay, days	4.4 (2–6)

Abbreviations: MI, myocardial infarction; UTI, urinary tract infection.

Note: Values are listed as number (percentage) or mean (range).

## Discussion

Acute calculus cholecystitis is a common cause of significant morbidity to a patient suffering from gallstone disease. It occurs most commonly due to an obstruction of the cystic duct by a gallstone leading to distension of the GB. As the GB becomes distended the blood flow is compromised resulting into local inflammation, mucosal ischemia, and finally necrosis. 15 In acute cholecystitis, surgery becomes more difficult as local inflammation may lead to bleeding, poor vision, and difficulty in dissection of the Calot's triangle. 16 As fibrosis progresses in the inflammatory process, adhesions may create a pull on the cystic duct leading to “tenting” of the CBD. 17 Traction applied to the Hartmann's pouch in the minimally invasive approach may further add to this tenting which can result into misidentification of the CBD as the cystic duct. The CBD can then be inadvertently clipped, lighted, or transacted with catastrophic consequences. BDI remains a significant problem and the major concern in the routine use of LC in cholecystitis. The risk of BDI is estimated to be between 0.26 and 0.7% of all LC 18 19 20 21 22 23 Grade 2 (moderate) cholecystitis doubled the risk while there is an eightfold increase in the risk of BDI in Grade 3 cholecystitis in one study. 24 Kum et al found a significant increase in BDI when LC was performed for acute cholecystitis when compared with elective LC (5.5 vs. 0.2%). 25 BDI can have disastrous consequences for the patient with major BDIs being associated with a 2 to 4% mortality rate. Long-term complications such as a biliary stricture can negatively affect the quality of life even years after the surgery and may require major surgical procedures like a bilioenteric bypass for correction. 6 7 8 26 27

So, when SAGES introduced its guidelines for LC in 1993, acute cholecystitis was mentioned as a relative contraindication. With advancement in minimally invasive technology and increased experience in laparoscopy, LC is increasingly being used for the management of acute cholecystitis. 28 29 30 Kiviluoto et al in a randomized trial showed that though LC in acute cholecystitis was technically demanding, it was safe, effective, and had lower complication rate compared with open cholecystectomy. 31 In 2018, the Tokyo Guidelines were updated to include LC in the management protocols of moderate and severe cholecystitis. 2 32

Many measures have been put forth to minimize intraoperative complications while performing LC for acute cholecystitis. Achieving the CVS prior to clipping of the cystic duct is of paramount importance and it remains the most commonly employed means of reducing BDI. Intraoperative cholangiogram (IOC) and other fluorescence imaging techniques to delineate the biliary anatomy are other methods to avoid iatrogenic injuries. There is considerable debate regarding use of IOC in LC. 17 20 33 IOC is technically challenging in the laparoscopic approach and may lead to an increased duration of surgery and trauma to the extrahepatic biliary tree during cannulation. There are also chances of an incorrect cannulation while working in an inflamed local environment and a distorted calot's triangle. IOC has therefore not gained universal acceptance and is reserved only when biliary injury is strongly suspected in routine practice. While use of ICG dye and a near infrared camera has well proven results, the need for new and expensive equipment makes the technology out of reach for many institutes in the developing world.

This study addresses the need of a safe and cost-effective method of safeguarding the CBD. The authors propose stenting the CBD preoperatively in cases of Grade 2 cholecystitis secondary to choledocholithiasis and using the stent as a guide during LC. The stent may provide a much-needed tactile feedback during dissection of an inflamed GB promoting the surgeon away from the CBD. It may help the surgeon plan the dissection and avoid going medial to the CBD. Once the calot's triangle is dissected fluoroscopic imaging may be used to identify the CBD with the stent in situ. Only after identification of the CBD, clipping of the cystic duct is performed. We recommend using a modern mobile c-arm with fluoroscopic and digitalized image capabilities as this equipment is readily available in most operating rooms. In the present study, we were able to correctly identify the CBD in all the cases despite severe local inflammation. The reported incidence of ERCP-induced pancreatitis is around 10% with a mortality of 0.7%. 34 In the majority of such cases, the diseases generally follow a benign course which is usually managed conservatively. On the other hand, the morbidity associated with a CBD injury is huge and long term, necessitating complex biliary reconstruction procedures in some cases. Therefore, the authors strongly feel that the benefits of biliary stenting to safeguard the CBD outweigh the risks associated with ERCP specially when performed by skilled endoscopists. The importance of our approach was highlighted on the follow-up ERCP where a gram showed no cystic duct stump leak, no long residual cystic duct, and no loss in the integrity of the extrahepatic biliary tree.

While there may be concerns regarding the potential inflammation and bacterial colonization caused by the placement of the stent, we found no increase in the duration of surgery or other surgical complication secondary to placement of a stent. Lee et al reported that the insertion of a stent was not a predictor of the use of a bailout procedure or a longer operative time. 35 In that study, biliary stenting did not lead to any increase in surgical complications. 36 Kawabata et al also experienced fewer surgical complications and a conversion rate of 2% when early LC was performed after prophylactic biliary stenting done for CBD stones. 37 In another study by Nair et al, they found short-term placement of CBD stent led to easier identification of the CBD during LC. 38 They also postulated that acute placement of CBD stent reduced the pressure in the biliary system by ensuring adequate bile drainage. 39 This may lead to a decrease in the postoperative biliary leaks. It was only when CBD stents were left in situ for prolonged periods of time that ensuing fibrosis distorted the anatomy of the calot's triangle which led to an increased conversion rate in one study. 38

Cholecystectomy was done using a laparoscopic fundus first or “top down” approach in three cases in the present study. In all the cases it was dense adhesions in the region of the calots triangle which prompted us to opt for a bail out procedure. The fundus was held and retracted inferiorly and with an open atraumatic grasper, the edge of the liver was retracted superiorly. The dissection was continued inferiorly while taking care not to injure the liver parenchyma. The hepatocytic triangle was then dissected and in all the cases, we were able to identify the CBD and cystic duct prior to clipping and there were no BDI reported. Conversion to open cholecystectomy was necessary in one case due to a frozen calots triangle. An incision was taken to connect the epigastric and the left working port and the abdominal wall was opened. A top down approach was used to dissect the GB. The CBD was identified by palpation of the previously placed stent. The cystic duct and cystic artery were then doubly ligated and cut. The postoperative course was uneventful in all these cases.

Lack of a control group and a small sample size limits the application of this study. It is a retrospective single-arm study where only the patients managed by a single experienced surgeon in a high-volume referral center were included for analysis. This may constitute a bias as it is unclear whether or not similar results would be produced by a less experienced surgeon. Larger, prospective, randomized trials are needed to confirm the utility of prophylactic CBD stenting in cases with acute cholecystitis to improve surgical outcomes. Further studies to evaluate the cost effectiveness of this approach are also recommended.

This technique can become a standard of care in all teaching institutions in developing country further enhancing the safety of cholecystectomy in gangrenous cholecystitis with a distorted biliary anatomy.

## Conclusion

In this study we presented our novel use of preoperative stenting the CBD and using intraoperative imaging technique for identification and safeguarding of the CBD while performing LC for an acute cholecystitis. This enabled us to correctly identify the biliary anatomy in all our cases and led to zero BDI. The advantage of this approach in minimizing BDI far outweighs the risks associated with biliary stenting and we propose this as a cost-effective alternative to the use of ICG dye and near infrared imaging in LC performed for acute cholecystitis.
